# Identification of genes differentially expressed during interaction of Mexican lime tree infected with "*Candidatus *Phytoplasma aurantifolia"

**DOI:** 10.1186/1471-2180-11-1

**Published:** 2011-01-01

**Authors:** Maryam Ghayeb Zamharir, Mohsen Mardi, Seyed Mohammad Alavi, Nader Hasanzadeh, Mojtaba Khayyam Nekouei, Hamid Reza Zamanizadeh, Ali Alizadeh, Ghasem Hoseini Salekdeh

**Affiliations:** 1 Department of Systems Biology, Agricultural Biotechnology Research Institute of Iran, Karaj, Tehran, Iran; 2 Department of Plant Disease, Faculty of Agriculture, Science and Research Branch, Islamic Azad University, Tehran, Iran; 3 Laboratory of Prokaryote, Department of Plant Disease, Iranian Research Institute of Plant Protection, Tehran, Iran; 4 Department of Genomics, Agricultural Biotechnology Research Institute of Iran, Karaj, Tehran, Iran; 5 Department of Molecular Systems Biology, Royan Institute, Tehran, Iran

## Abstract

**Background:**

*"Candidatus *Phytoplasma aurantifolia", is the causative agent of witches' broom disease in Mexican lime trees (*Citrus aurantifolia *L.), and is responsible for major losses of Mexican lime trees in Southern Iran and Oman. The pathogen is strictly biotrophic, and thus is completely dependent on living host cells for its survival. The molecular basis of compatibility and disease development in this system is poorly understood. Therefore, we have applied a cDNA- amplified fragment length polymorphism (AFLP) approach to analyze gene expression in Mexican lime trees infected by "*Ca*. Phytoplasma aurantifolia".

**Results:**

We carried out cDNA-AFLP analysis on grafted infected Mexican lime trees of the susceptible cultivar at the representative symptoms stage. Selective amplifications with 43 primer combinations allowed the visualisation of 55 transcript-derived fragments that were expressed differentially between infected and non-infected leaves. We sequenced 51 fragments, 36 of which were identified as lime tree transcripts after homology searching. Of the 36 genes, 70.5% were down-regulated during infection and could be classified into various functional groups. We showed that Mexican lime tree genes that were homologous to known resistance genes tended to be repressed in response to infection. These included the genes for modifier of snc1 and autophagy protein 5. Furthermore, down-regulation of genes involved in metabolism, transcription, transport and cytoskeleton was observed, which included the genes for formin, importin β 3, transducin, L-asparaginase, glycerophosphoryl diester phosphodiesterase, and RNA polymerase β. In contrast, genes that encoded a proline-rich protein, ubiquitin-protein ligase, phosphatidyl glycerol specific phospholipase C-like, and serine/threonine-protein kinase were up-regulated during the infection.

**Conclusion:**

The present study identifies a number of candidate genes that might be involved in the interaction of Mexican lime trees with "*Candidatus *Phytoplasma aurantifolia". These results should help to elucidate the molecular basis of the infection process and to identify genes that could be targeted to increase plant resistance and inhibit the growth and reproduction of the pathogen.

## Background

*"Candidatus *Phytoplasma aurantifolia" is an obligate biotrophic plant pathogen that causes witches' broom disease in Mexican lime trees (*Citrus aurantifolia *L.). This is a devastating disease that results in significant economic losses [1]. Phytoplasmas are prokaryotes that inhabit the phloem and are transmitted by phloem-sucking insects [2,3]. It has been demonstrated that "*Ca*. Phytoplasma aurantifolia" is transmitted by the leafhopper *Hishimonus phycitis *(Hemiptera: Cicadellidae) [4].

The mechanisms that regulate the distribution of phytoplasmas in the host tissue is still widely unknown. At the tissue level, phytoplasma infection can cause anatomical aberrations such as extensive necrosis the phloem and phloem tissue proliferation, which results in swollen veins [5]. Reduced stomatal conductance has also been observed, together with impaired photosynthesis [6]. The genomes of the phytoplasmas are extremely reduced and many genes that encode components of essential metabolic pathways in other organisms are missing. It is likely phytoplasmas are unable to synthesize nucleotides and need to import them from the host plant. Important genes encode for enzymes involved in the biosynthesis of amino acids and fatty acids are also missing. In addition, because phytoplasmas are the only known organisms without an ATP-synthase, they probably need to import ATP from the environment as well [5,7,8]. This highly specialised nutritional requirements, which typifies biotrophic plant pathogens such as phytoplasmas, probably involves the strict control of host cell metabolism which is diverted to maintain a suitable environment for the pathogen [9].

The molecular details of the infection process are largely unknown. Initial details were obtained from studies of phytoplasma/plant interactions with respect to polyphenol production and the transport of sugar and amino acid and comprehensive differences in gene expression have reported mainly in the experimental host plant periwinkle (*Catharanthus roseus *L.) [10,11]. However, molecular data from the direct investigation of compatible interactions in cultivated Mexican lime tree genotypes are scarce, and witches' broom disease has received little attention as compared with diseases carried by other phytoplasma pathogens, such as Aster yellows phytoplasma [9]. In this study, we applied a cDNA- amplified fragment length polymorphism (AFLP) approach to identify genes that may be expressed differentially in Mexican lime trees infected with "*Ca*. Phytoplasma aurantifolia". Understanding the basis of susceptibility to the pathogen will assist greatly in the development of new control strategies and the identification of pathogen and host factors that are required for disease progression.

## Results

**Figure 1:**
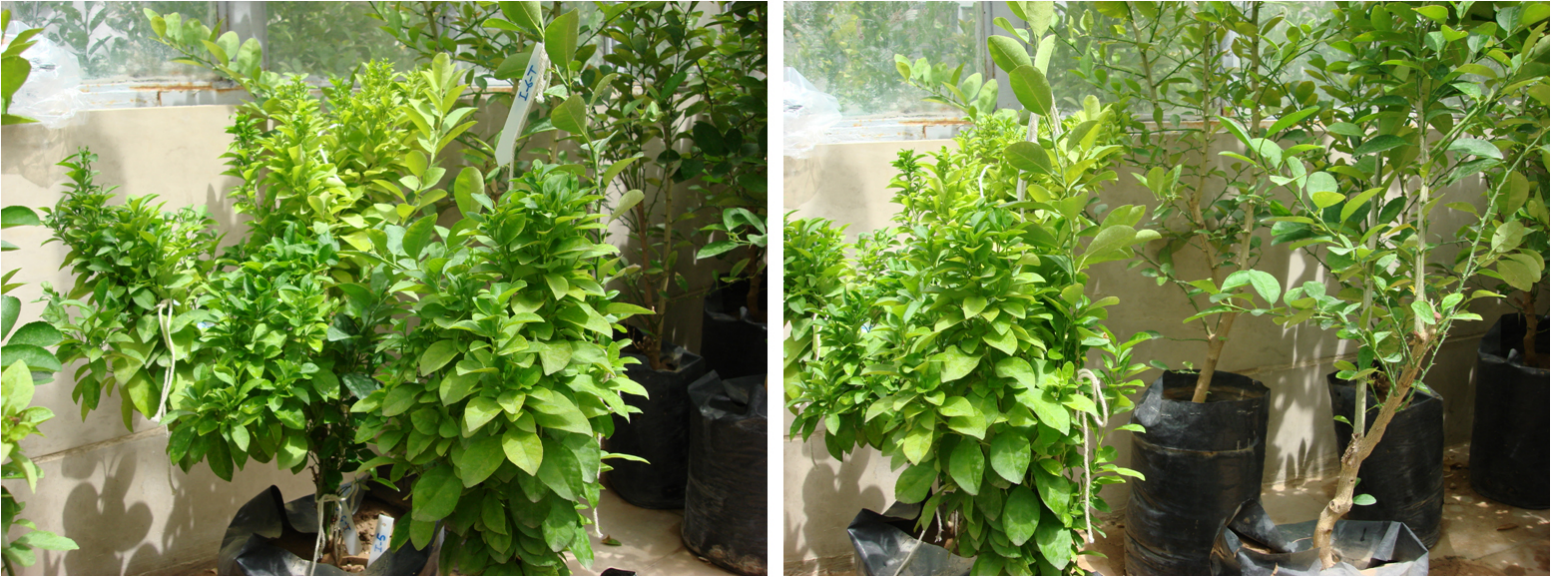
**Healthy and infected plants**. Approximately 5 months after diseased branches were grafted onto healthy Mexican lime trees (right of figures), the plants developed typical symptoms of witches' broom disease (left of figures).

Five months after grafting healthy Mexican lime trees, plants developed the typical symptoms of witches' broom (Figure 1). The results of nested PCR further confirmed the incidence of phytoplasma infection in grafted plants (Additional File 1). Analysis with iPhyClassifier revealed that the virtual restriction fragment length polymorphism (RFLP) pattern that was derived from the phytoplasma 16 S rDNA fragment amplified from the diseased specimens was most similar to the reference pattern of the 16Sr group II, subgroup B phytoplasma (GenBank accession: U15442), with a pattern similarity coefficient of 0.99. Therefore, the phytoplasma under study was a variant of 16SrII-B and related to "*Ca*. Phytoplasma aurantifolia".

### cDNA-AFLP analysis

**Figure 2:**
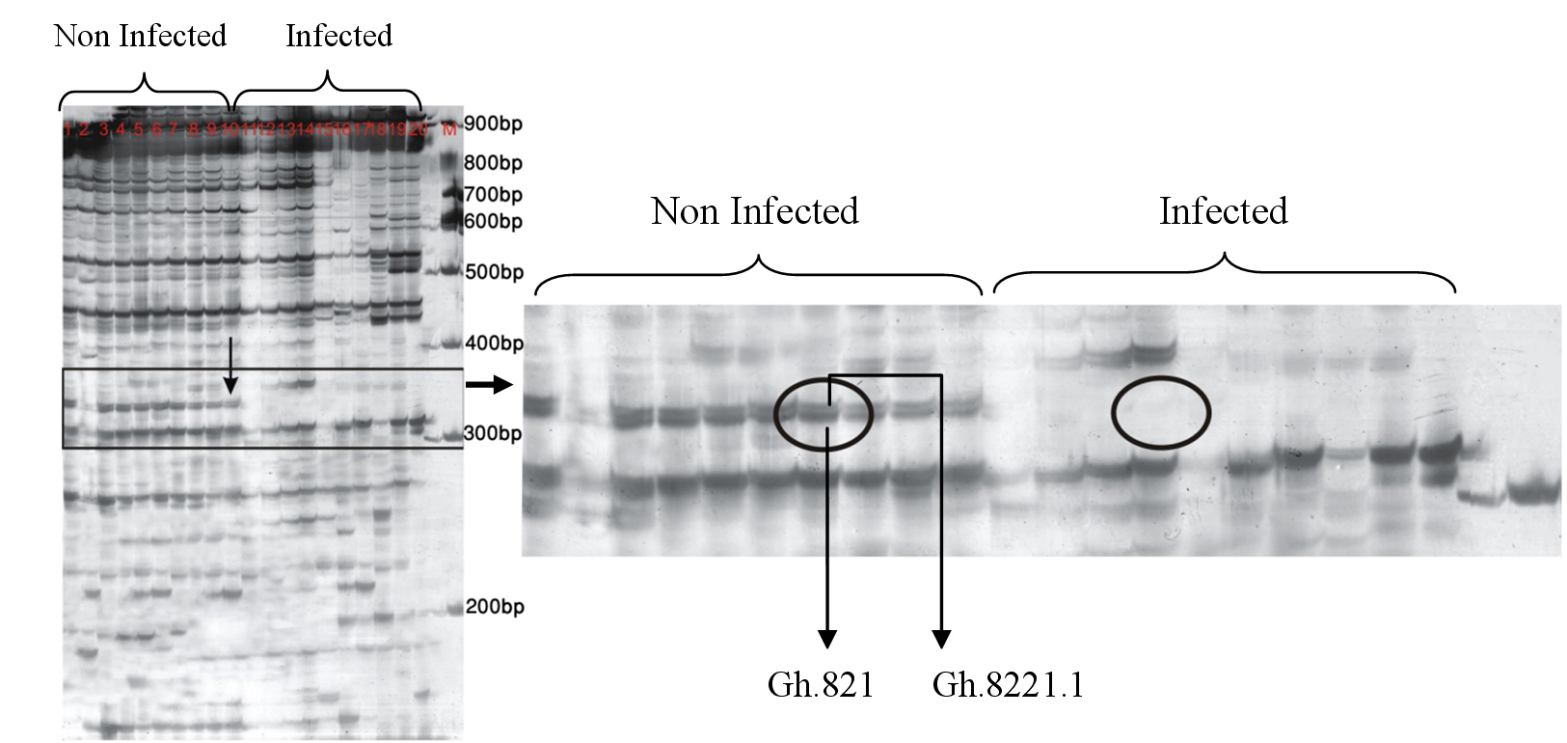
**Representative results of polyacrylamide gel of cDNA-AFLPs generated by the primer combinations E11/MCG**. Wells 1-10, 11-20, and M present non-infected, infected and 100 bp DNA size marker, respectively. Gh.821 and Gh.8221.1 represent two differentially expressed transcript derived fragments (DE-TDFs) that were identified as autophagy protein 5.

For each of the 43 primer combinations, 40-100 different transcript derived fragments (TDFs), which ranged from 50 to 800 bp, were visualized as bands (Figure 2).

Analysis of the expression profiles of the infected and noninfected samples between replicates revealed 55 differentially expressed TDFs (DE-TDFs) that showed the same pattern in all replicates. Fifty-one of these DE-TDFs were isolated and sequenced. The remaining four DE-TDFs could not be cloned and were excluded from analysis. Out of the 51 sequenced DE-TDFs, 36 showed similarity to known gene sequences in databases (Table 1), whereas 15 DE-TDFs did not show homology to any known nucleotide or amino acid sequences. All 51 TDFs sequences were submitted to the NCBI database with accession numbers assigned and reported in Table 1.

**Table 1:** Homologies of the transcript derived fragments (TDFs) to known sequences in the databases.

TDF	Length (bp)	Accession number	I/R	Annotation (plant, accession number)	E-value
**Stress response/defense**					
Gh16122	444	GT222039	I	Proline-rich protein (*Cladrastis kentukea*, AAG15241.1)	1e-12
Gh11114	158	GT222037	R	Modifier of snc1 (*Ricinus communis*, XP_002522998.1)	6e-04
Gh11112	157	GT222036	R	Modifier of snc1 (*Ricinus communis*, XP_002522998.1)	6e-4
Gh921	191	GT222045	R	Autophagy protein 5 (*Glycine max*, AM087008.1)	3e-19
Gh8221.1	198	GT222040	R	Autophagy protein 5 [*Glycine max*, AM087008.1)	5e-08
Gh8221.2	190	GT222035	R	Autophagy protei n (*Glycine max*, AM087008.1)	4e-19
Gh821	191	GT222047	R	Autophagy protein 5 (*Glycine ma*x AM087008.1)	1e-29
Gh542	316	GT222056	I	hypothetical protein with lysine domain (*Medicago sativa*, XP_002278178.1)	3e-22
Gh7111	69	GT222032	I	Serine-rich protein-related, *Cichorium intybus*, TA1423_13427	7e-51
Gh16121	162	GT222038	I	Serine-rich protein-related, *Cichorium intybus*, TA1423_13427	1e-49
**Cell Metabolism**					
Gh1574	526	GT222018	I	Phosphatidyl glycerol specific phospholipase C-like (Sweet orange, EY651478.1)	1e-40
Gh511	113	GT222066	R	L-asparaginase (*Ricinus communis*, ref-XM_002510114.1)	5e-06
Gh7123	263	GT222042	R	Glycerophosphoryl diester phosphodiesterase (*Ricinus communis*, XP_002512887.1)	4e-27
Gh532	181	GT222058	R	Retroelement pol polyprotein-like (*Arabidopsis thaliana *, BAB10790)	1e-14
**Protein synthesis/destination**					
Gh1633	416	GT222024	I	50 S ribosomal protein L15 (*Ricinus communis*, XP_002531621.1)	2e-19
Gh1631	416	GT222023	R	50 S ribosomal protein L15 (*Ricinus communis*, XP_002531621.1)	5e-18
Gh553-2	323	GT222065	R	Ubiquitin-protein ligase *(Vitis vinifera*, XM_002305323.1)	4e-09
Gh553-1	181	GT222064	R	Ubiquitin-protein ligase *(Vitis vinifera*, XM_002305323.1	1e-11
**Signal transduction Protein**					
Gh1822	161	GT222030	R	Transducin family protein (*Arabidopsis thaliana*, NM_180281.2)	5e-15
Gh1821	160	GT222029	R	Transducin family protein (*Arabidopsis thaliana*, NM_180281.2)	4e-16
Gh324	241	GT222061	I	Serine/threonine-protein kinase (*Ricinus communis*, XM_002531749.1)	3e-15
**Transporter**					
Gh1572	380	GT222017	I	Similar to Importin β 3 (Citrus clementina, DY277746)	2e-13
Gh1521	402	GT222015	I	Similar to Importin β 3 (Citrus clementina, DY277746)	5e-14
**Transcription**					
Gh1591	722	GT222019	R	RNA polymerase beta' gene, partial cds; chloroplast. (*Citrus sinensis*, YP_740466.1)	2e-139
**Cytoskeleton**					
Gh1811	148	GT222028	R	Formin (*Ricinus communis*, XP_002532961.1)	2e-04
**Unknown function**					
Gh7101	108	GT222044	R	Root salinity induced TDF *(Spartina alterniflora*, DR010701.1)	3e-05
Gh1511	123	GT222014	R	Poncirus trifoliata Roots with Iron Deficiency, CX640377.1)	2e-06
Gh521	195	GT222059	R	subtracted infection mimic Phytophthora infestans cDNA (CV945240.1)	4e-121
Gh1623	119	GT222021	R	Leaf infected with Xylella fastidiosa (Sweet orange, EY666062.1)	3e-09
Gh821	191	GT222047	R	Development (Citrus sinensis cDNA, EY722243.1)	1e-29
Gh1661	203	GT222025	R	Phloem Citrus sinensis cDNA clone (*Citrus sinensis*, DR910976.1)	2e-74*
Gh1624	589	GT222022	R	Mexican lime leaf, greenhouse plant, EY854330.1	8e-08
Gh721	110	GT222054	R	root salinity induced TDF (*Spartina alterniflora*, DR010701.1)	3e-09
Gh541	308	GT222057	R	Plant transcript (*Citrus sinensis*, TA16449_2711)	2e-50
Gh543	314	GT222055	I	Slow drought stressed root cDNA library (*Cicer arietinum*, GR410097.1)	6e-122
Gh734	36	GT222052	R	Slow drought stressed root cDNA library (*Cicer arietinum*, GR410033.1)	6e-122

TDFs were cloned and sequenced from one health (R; repressed) or infected (I; induced) plant.

### Gene ontology analysis of Mexican lime tree transcripts modulated by witches broom infection

**Figure 3:**
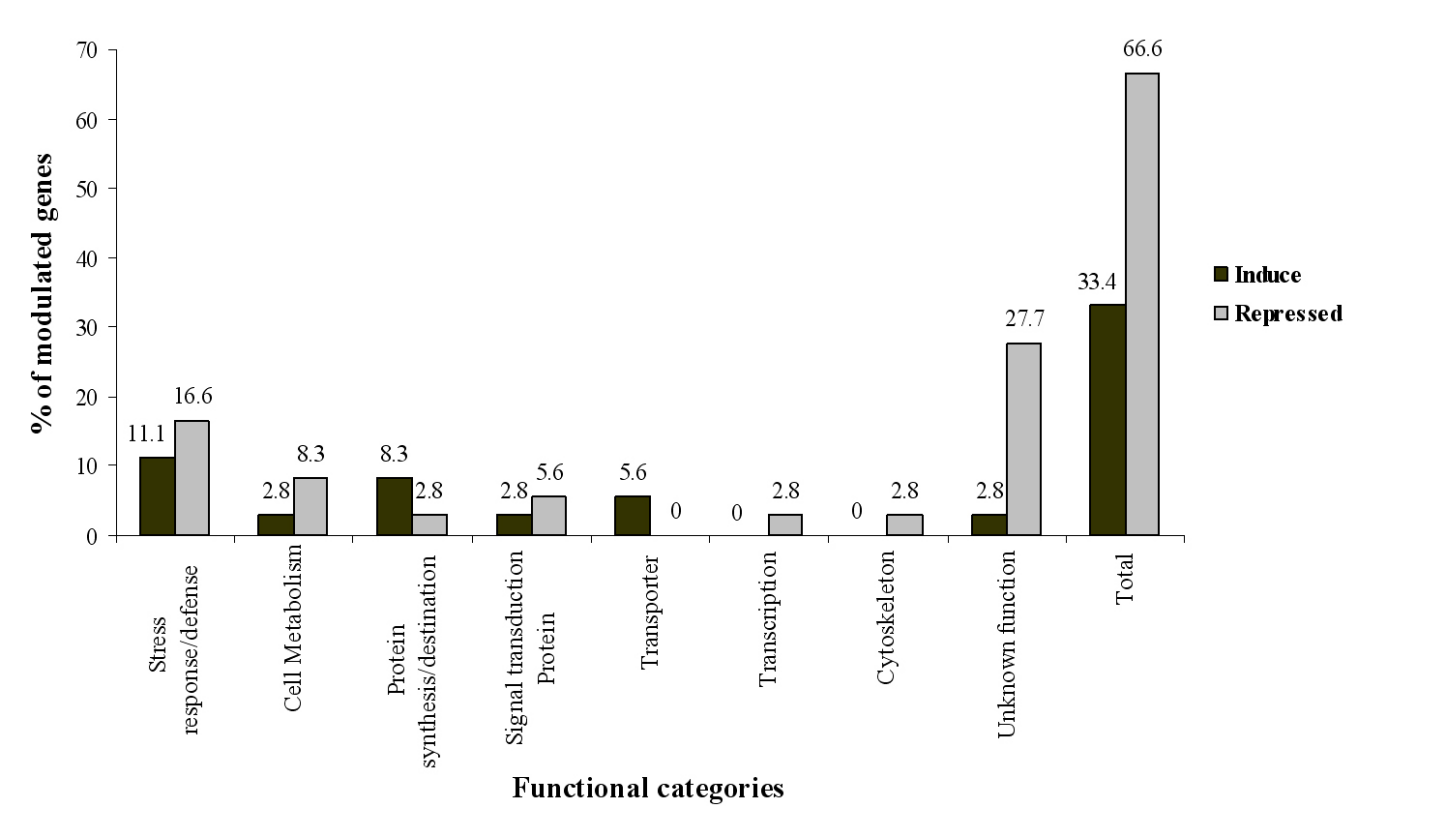
**Functional classification**. Functional categories of transcripts modulated by "*Ca*. Phytoplasma aurantifolia".

Each of the 51 sequenced transcript was annotated functionally through careful analysis of the scientific literature and the Gene Ontology Databases. Out of 51 sequenced DE-TDFs, 36 (80%) could be assigned to one of the following functional groups: stress response/defense (10 TDFs), cell Metabolism (4 TDFs), protein synthesis/destination (4 TDFs), signal transduction (3 TDFs), transporter (2 TDFs), transcription (1 TDFs), cytoskeleton (1 TDFs) and unknown function (11 TDFs). The molecular function of each individual protein is given in Table 1. The stress response/defence group contained 27.7% of the DE-TDFs and constituted the largest functional group (Figure 3).

### Verification of representative genes by real-time RT-PCR

**Figure 4:**
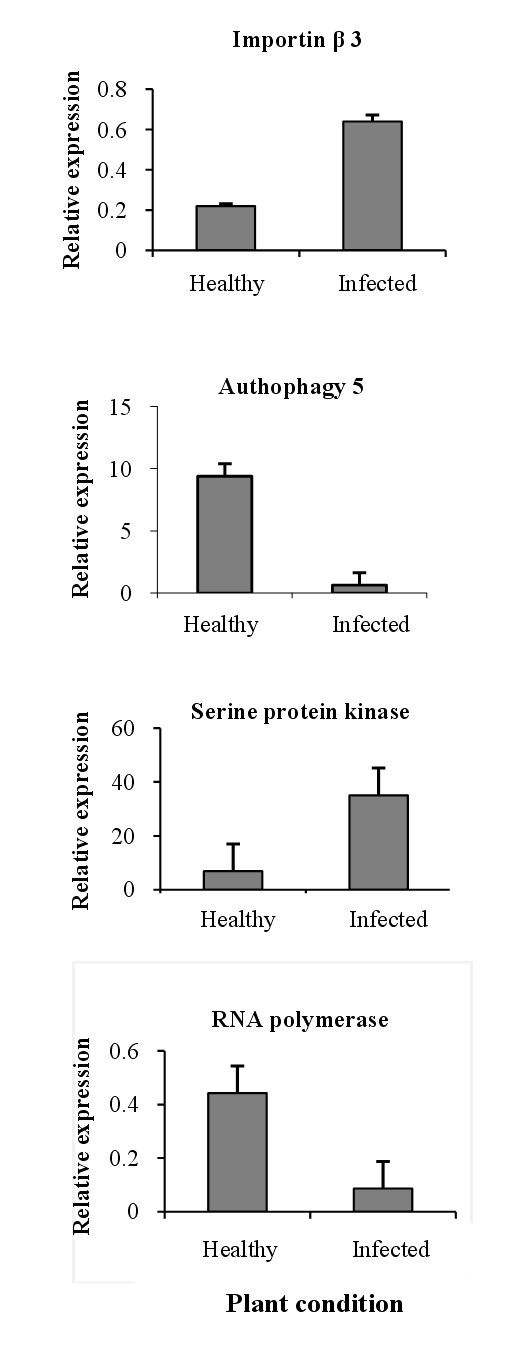
**Real-time analysis of four differentially expressed transcript derived fragments (DE-TDFs)**. The Y axis represents the relative expression (expression normalised to that of the housekeeping gene).

To verify the expression patterns that were identified in the cDNA-AFLP study, the expression level of four DE-TDFs was analyzed by real-time RT-PCR. These TDFs were chosen to represent the various functional categories identified (Table 1). Two of the selected TDFs (serine/threonine-protein kinase and importin β) were more abundant in infected plants, whereas two TDFs (autophagy protein 5 and RNA polymerase β) showed higher expression in healthy plants. The 18 s RNA gene of Mexican lime tree was used as a reference gene for data normalization, as described previously [12]. Real-time PCR analysis showed that the expression of the selected genes agreed well with the profiles determined by cDNA-AFLP (Figure 4).

## Discussion

In this study, we performed a comparative transcriptomic analysis of healthy Mexican lime trees and those infected by "*Ca*. Phytoplasma aurantifolia" by using cDNA-AFLP technique. For this analysis, we used leaf samples from healthy controls and infected plants at the symptomatic stage. The symptomatic stage was chosen because the plant/pathogen interaction is well established but the plant cells are still active and can maintain pathogen survival.

As far as we are aware, our study is the first gene expression analysis of the compatible interaction between "*Ca*. Phytoplasma aurantifolia" and Mexican lime trees. We observed transcriptional changes that affected the expression of several genes related to physiological functions that would affect most leaves in infected tissues.

The cDNA-AFLP method for global transcriptional analysis is an open architecture technology that is appropriate for gene expression studies in non-model species. This is because prior sequence data are not required for the visual identification of differentially-expressed transcripts, in contrast to other approaches.

### Infection with "*Ca*. Phytoplasma aurantifolia" causes widespread gene repression in Mexican lime trees

Sixty-seven percent of the identified DE-TDFs were down-regulated in response to infection, whereas only 33% were up-regulated in response to infection which could reflect the exploitation of cellular resources and the suppression of defence responses by the phytoplasma [13].

### Responses to external stimuli and defence

Several genes that were modulated in Mexican lime trees by infection with "*Ca*. Phytoplasma aurantifolia" were related to defence, cell walls, and response to stress. The expression of autophagy protein 5 was repressed. Autophagy is a survival mechanism that protects cells against unfavourable environmental conditions, such as microbial pathogen infection, oxidative stress, nutrient starvation, and aggregation of damaged proteins [14]. It has been shown that carbohydrate starvation induces the expression of autophagy genes [15] and stimulates the formation of reactive oxidative species (ROS) in plants [14]. Biochemical analyses on phytoplasma-infected tobacco plants have indicated that soluble carbohydrates and starch were accumulated in source leaves, whereas sink organs showed a marked decrease in sugar levels. Similar changes in carbohydrate metabolism have been described in coconut palms infected with the lethal yellowing phytoplasma [16]. It is likely that the accumulation of carbohydrate reduces the expression of autophagy genes in the host and limits the burst of ROS burst (hypersensitivity reaction). These effects might result in reduced host resistance to phytoplasma and create a suitable conditions for phytoplasma survival in the host.

We also identified a cell wall hydroxyl proline-rich protein (GT222039) that was induced in response to the pathogen. Proline-rich proteins are among the major structural proteins of plant cell walls. Environmental stresses can alter the composition of the plant cell wall markedly [17]. It has been demonstrated that mechanical wounding, infection, or elicitors obtained from microbial cell walls or culture fluids caused accumulation of specific hydroxyl proline-rich glycoproteins and other antimicrobial cell wall proteins [17]. It has been reported that elicitors cause an H_2_O_2_-mediated oxidative cross-linking of preexisting structural cell wall proteins that precedes the activation of transcription-dependent defences. The induction of the hydroxyl proline-rich protein in the present study might reflect a defence mechanism of Mexican lime tree in response to phytoplasma infection.

Another induced protein (GT222056) contained a lysine domain that is found in several enzymes that are involved in degradation of the bacterial cell wall [18]. The role of this gene in the response of Mexican lime trees to the pathogens remains to be determined.

Two of repressed genes (GT222036 and GT222036) were identified as a modifier of snc1 (MOS1). Plant resistance (R) genes encode immune receptors that recognise pathogens directly or indirectly and activate defence responses [19]. The expression levels of R genes have to be regulated tightly due to costs to the fitness of plants that are associated with maintaining R-protein-mediated resistance. Recently, it has been reported that MOS1 regulates the expression of SNC1 which encodes a TIR-NB-LRR-type of R protein in Arabidopsis.

It has been shown that mos1 mutations reduce the expression of endogenous snc1, which results in the repression of constitutive resistance responses that are mediated by snc1 [20]. It is likely that down-regulation of Mexican lime tree MOS1 in response to the pathogen reflects a reduction in plant resistance responses to phytoplasma infection.

### Cell Metabolisms

Lipid-derived molecules act as signals in plantpathogen interactions, and the roles of jasmonic acid and related oxylipins that are produced from membrane-derived fatty acids through beta-oxidation, are particularly important [21]. During infection, low level defence responses can be activated in susceptible plants [22,23]. Therefore, it is likely that well-established "*Ca*. Phytoplasma aurantifolia" infections involve the up-regulation of genes that encode components of the lipid metabolism pathway, such as phosphatidyl glycerol specific phospholipase C-like (GT222018). This enzyme regulates the phosphatidylglycerol content via a phospholipase C-type degradation mechanism [24]. Another gene involved in lipid metabolisms, glycerophosphoryl diester phosphodiesterase (GT222042) was repressed during the infection. This enzyme has both phosphoric diester hydrolase and glycerophosphodiester phosphodiesterase activity and is involved in the metabolism of glycerol and lipids [25].

### Protein synthesis and destination

We identified several TDFs that were related to protein metabolism in our study. Among these were genes that encoded ribosomal proteins and enzymes involved in degradation. The expression of two ubiquitin-protein ligases (GT222065 and GT222065) and one 50 S ribosomal protein L15 (GT222023) were repressed, whereas another 50 S ribosomal protein L15 (GT222024) was induced. This suggests that the infection results in a general induction of protein turnover, which could reflect an adaptive response in the plants to remove misfolded proteins that have accumulated as a result of stress [23].

### Signal transduction

Three of the modulated genes had signal transduction and/or gene regulation functions. They corresponded two transducin family protein (GT222030 and GT222029) that were repressed by infection and a serine/threonine protein kinases (GT222061) that was induced during infection. Serine/threonine protein kinases are a group of enzymes that catalyse the phosphorylation of serine or threonine residues in proteins, with ATP or other nucleotides acting as phosphate donors. The phosphorylation of proteins on serine, threonine, or tyrosine residues is an important biochemical mechanism to regulate the activity of enzymes and is used in many cellular processes [26].

The two down-regulated proteins were identified as members of the transducin family and contained WD40 domain. This domain is found in several eukaryotic proteins that with wide variety of functions, which include adaptor/regulatory modules in signal transduction, together with proteins involved in pre-mRNA processing, and cytoskeleton assembly [27]. It is unclear how these changes contribute to the response of Mexican lime tree to infection.

## Conclusion

We believe that this study is the first reported analysis of the expression of genes involved in the interaction of Mexican lime trees with "*Ca*. Phytoplasma aurantifolia". The cDNA-AFLP technique allowed several novel genes to be identified from Mexican lime trees, because a significant proportion of the TDFs are not currently represented in citrus databases. Our data showed that infection resulted in the down-regulation of Mexican lime tress transcripts in all major functional categories. However, certain genes that were required for plant-pathogen interactions were modulated positively during infection at the symptomatic stage. These results will serve as a basis to address new questions and design new experiments to elucidate the biology of plant-phytoplasma interactions and the associated re-programming of the host metabolism. They might also pave the way to identify genes that can be targeted to elevate plant resistance or inhibit the growth and reproduction of the pathogen. However, further research is required to elucidate the roles of these genes in the susceptibility/resistance of Mexican lime tress to "*Ca*. Phytoplasma aurantifolia", and to determine how strategies might be developed to incorporate these genes into molecular breeding programmes.

## Methods

### Plant material and inoculation

Ten healthy 1-year-old Mexican lime trees grown in the greenhouse were used in this experiment. Specimens from Mexican lime trees infected with witches' broom were grafted to healthy trees, and specimens from healthy Mexican lime trees were grafted to other healthy trees. The grafted plants were covered for 1 month with plastic bags to increase humidity and were arranged randomly on the greenhouse bench. They were kept under natural light conditions at a temperature of 25-28°C. The branches infected with witches' broom were sampled 20 weeks after inoculation and used for RNA extraction. As a control, RNA was extracted from non-grafted healthy plant leaves that has been grown under similar conditions.

### Detection of Phytoplasma infection by nested PCR

Total DNA was extracted from leaf samples (vascular tissues from leaf veins and petioles) using the method described originally by Daire *et al *[28] with some modifications [29]. Samples of tissue (1 g) were homogenised at room temperature in 7 ml of cetyl trimethyl ammonium bromide (CTAB) buffer (3% CTAB, 1 M Tris-HCl pH 8.2, mM EDTA, 1.4 M NaCl), with addition of 0.2% 2-mercaptoethanol, in disposable plastic bags using a ball-bearing device. Aliquots of 1 ml of homogenate were transferred to Eppendorf tubes and incubated in a water bath at 65°C for 20 min. After extraction with 1 ml of chloroform, nucleic acids were precipitated from the aqueous phase with an equal volume of isopropanol, collected by centrifugation, washed with 70% ethanol, dried, dissolved in 150 ml of TE buffer (10 mM Tris, 1 mM EDTA, pH 7.6) and stored at -20°C until use. The region of the phytoplasma 16 S rRNA gene was amplified by PCR in a total reaction volume of 25 μl in an Applied Biosystems thermal cycler. The first set of PCR primers was P1 (5'-AAGAGTTTGATCCTGGCTCAGGATT-3') [30] and P7 (5'-CGTCCTTCATCGGCTCTT -3') [31]. The resulting P1-P7 amplicons were then used as template DNA in a nested-PCR amplification with the universal primer pair for phytoplasmas r16r2/r16F2n [32]. The purified PCR products were cloned into the pGEM-T Easy vector (Promega), and sequenced at the fluorescent automated sequencing facility at Fazabiotech (Tehran, Iran). The phytoplasma strains were classified using iPhyClassifier, as described by Zhao *et al *[33].

### RNA isolation and cDNA synthesis

Total RNA was extracted from 5 phytoplasma-infected Mexican lime trees and 5 healthy trees using Trizol reagent (Invitrogen, Life Technologies) in accordance with the manufacturer's instructions. cDNA synthesis and cDNA-AFLP analysis were performed for the 10 replicates. First-strand cDNA was synthesised from 2 μg of total RNA using a SuperScript III First Strand Synthesis System (Invitrogen, USA) in accordance with the manufacturer's instructions. Second-strand cDNA was sythesised by adding the first-strand cDNA reaction to a reaction mix that contained 15 μl of 10 × cDNAII buffer, 35 U DNA of Polymerase I (Invitrogen), 3 U of RNase H (Invitrogen), and 1 μl dNTPs (25 mM) in a final volume of 150 μl, and incubating for 2 h at 16°C (). The resulting double-stranded cDNA was purified in accordance with the method of Powell and Gannon [34]. The concentration of the cDNAs was determined using spectrophotometer (Bio-Rad) and their quality was determined by electrophoresis on a 1.2% agarose gel.

### cDNA- AFLP

A 500-ng aliquot of double-stranded cDNA was used for AFLP analysis as described by Bachem *et al*. [35] with the following modifications. The template for cDNA-AFLP was digested with the restriction enzymes, *EcoR*I/*Mse*I and *Psu*I/*Mse*I (Invitrogen). The Sequence of the primers and adapters used for the AFLP reactions are given in Additional File 2. AFLP reactions were performed in accordance with Bachem *et al*. [36]. Selective amplification products were separated on a 10% polyacrylamide gel and stained with silver nitrate [37]. The gels were dried onto 3 MM Whatman paper.

### Cloning, sequencing and bioinformatic characterisation

To select DE-TDFs, the profiles of infected and non-infected samples were compared between replicates. TDFs that differed in abundance between the two types of sample, namely infected and non-infected plants, were selected only when the same pattern was observed in all replicates.

The cloning of bands of interest was performed as previously described[38]. Briefly, the bands were excised from the gels using a razor blase. Each gel slice was incubated in 10 μl of distilled water for 10 min at 96°C. Aliquots of the eluent were subjected to PCR using the same conditions as for the selective PCR described before. PCR products were separated on 10% polyacrylamide gel to confirm that the correct polymorphic fragments had been selected [39].

After verification, the recovered products re-amplified using primer pair E-0/M-0 and P-0/M-0 to provide sufficient DNA for cloning. The purified PCR products were cloned into the pGEM-T Easy vector (Promega) and then sequenced.

The sequences were compared with those in the non-redundant databases of the National Center for Biotechnology Information (NCBI; http://www.ncbi.nlm.nih.gov/BLAST/) and The Arabidopsis Information Resource (TAIR; http://www.arabidopsis.org/Blast/) using the Blastn, Blastx, and tBlastx sequence alignment algorithms [40]

### Real-time RT-PCR analysis

Real-time RT-PCR was carried out using RNA derived from infected and non-infected Mexican lime trees as described previously by Martini *et al*. [41]. Aliquots of each RNA sample (2 μl) were used to synthesise cDNA using a SuperScript III First Strand Synthesis System (Invitrogen). Primer pairs were designed using OligoTech Software (Additional File 3). Gene expression was assayed using the iCycler iQ, Multicolor Real-Time PCR Detection System (Bio-Rad) and iQ SYBR Green Supermix kit (Bio-Rad). Reaction conditions (20 μl volumes) were optimized by changing the primer concentration and annealing temperature to minimise primer-dimer formation and increase PCR efficiency. The following PCR profile was used: 2 min at 95°C, (95°C for 20 s, 60-63°C for 20 s, 72°C for 20 s) × 45, and 1 min at 72°C followed by recording of a melting curve. The absence of primer-dimers or accumulation of nonspecific products was checked by melting-curve analysis. Each run included standard dilutions and negative reaction controls. The expression levels of each gene of interest and of the 18 S rRNA, which was chosen as a housekeeping gene, were determined in parallel for each sample. Results were expressed as the ratio of the mRNA level of for each gene of interest normalised over housekeeping gene using the difference between threshold cycle values or ΔΔCt method [42,43]. Ct values for individual target genes were calculated and the ΔCt average for the housekeeping gene was treated as an arbitrary constant and used to calculate ΔΔCt values for all samples. The mean fold induction for the three independent pools for each target gene was determined and the standard error of the mean was calculated. The list of primers used for the real-time PCR analysis is presented in Additional File 3.

## Authors' contributions

MGZ carried out the cDNA-AFLP experiments (including the extraction and reamplification of cDNA fragments) participated in sequence analysis, performed the real-time RT-PCR experiments, and contributed to data interpretation and manuscript writing. MM participated in in the analysis and interpretation of cDNA-AFLP data. SMA participated in plant sample preparation. NHZ, HRZ, and AA participated in sequence analysis, in interpretation of data, in automatic and Gene Ontology assignment. GHS conceived the study, participated in its design and coordination, participated in interpretation of the data, in manual ontology assignments and wrote the manuscript. All authors read and approved the final manuscript.

## Supplementary Material

Additional File 1 **Agarose gel electrophoresis of nested PCR product from Mexican lime tree infected by "*Ca*. Phytoplasma aurantifolia" and from healthy plants**. Click here for file

Additional File 2 **Primer sequences used for cDNA AFLP analysis**. Click here for file

Additional File 3 **Primer sequences used for Real-Time PCR analysis**. Click here for file
