# Supplementary material for: Identification of genes differentially expressed during interaction of Mexican lime tree infected with "Candidatus Phytoplasma aurantifolia"
Source: BMC Microbiol. 2011 Jan 1;11:1. doi: 10.1186/1471-2180-11-1 (PMC3271359; doi:10.1186/1471-2180-11-1)
Supplement: Additional File 1 — Agarose gel electrophoresis of nested PCR product from Mexican lime tree infected by "Ca. Phytoplasma aurantifolia" and from healthy plants. [file 1471-2180-11-1-S1.docx]

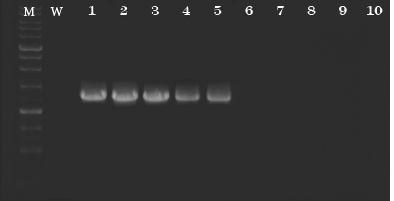


Additional File 1. Agarose gel electrophoresis of nested PCR products from Mexican lime trees infected by “*Ca.* Phytoplasma aurantifolia” (1-5) and from healthy plants (6-10). W is water and M is 1 Kbp DNA ladder.
