# Supplementary material for: Identification of genes differentially expressed during interaction of Mexican lime tree infected with "Candidatus Phytoplasma aurantifolia"
Source: BMC Microbiol. 2011 Jan 1;11:1. doi: 10.1186/1471-2180-11-1 (PMC3271359; doi:10.1186/1471-2180-11-1)
Supplement: Additional File 2 — Primer sequences used for cDNA AFLP analysis. [file 1471-2180-11-1-S2.docx]

Additional File 2. Primer sequences used for cDNA-AFLP analysis

| Primer Name | **Number of Nucleotides** | **Sequence** |
| --- | --- | --- |
| Ead-1 | 17 mer | 5′-CTC Gta GAC TGC GTA CC-3′ |
| Ead-2 | 18 mer | 5′-AAT TGG TAC GCA GTC TAC-3′ |
| Mad-1 | 16 mer | 5′-GAC GAT GAG TCC TGA G-3′ |
| Mad-2 | 14 mer | 5′-TAC TCA GGA CTC AT-3′ |
| E-000 | 16 mer | 5′-GAC TGC GTA CCA ATT C-3′ |
| M-000 | 16 mer | 5′-GAT GAG TCC TGA GTA A-3′ |
| P-000 | 21 mer | 5-GAC TGC GTA CAT GCA GCT CA-3´ |
| E-46 | 19 mer | 5′-GAC TGC GTA CCA ATT CGT C-3′ |
| E-11 | 19 mer | 5′-GAC TGC GTA CCA ATT CAG G-3′ |
| E-2 | 19 mer | 5′-GAC TGC GTA CCA ATT CAA C-3′ |
| E-GTT | 19 mer | 5′-GAC TGC GTA CCA ATT CGTT-3′ |
| E-TG | 18 mer | 5′-GAC TGC GTA CCA ATT CTG-3′ |
| E-GAC | 19 mer | 5′-GAC TGC GTA CCA ATT CGA C-3′ |
| E-TAT | 19 mer | 5′-GAC TGC GTA CCA ATT CTA T-3′ |
| M-c | 19 mer | 5′-GAT GAG TCC TGA GTA AC -3′ |
| M-a | 19 mer | 5′-GAT GAG TCC TGA GTA AA -3′ |
| MAC | 18 mer | 5′-GAT GAG TCC TGA GTA AAC-3′ |
| MGA | 18 mer | 5′-GAT GAG TCC TGA GTA AGA -3′ |
| MTG | 18 mer | 5′-GAT GAG TCC TGA GTA ATG-3′ |
| MGGG | 19 mer | 5′-GAT GAG TCC TGA GTA AGG G-3′ |
| MAAA | 19 mer | 5′-GAT GAG TCC TGA GTA AAA A-3′ |
| MCG | 18 mer | 5′-GAT GAG TCC TGA GTA ACG-3′ |
| MTC | 18 mer | 5′-GAT GAG TCC TGA GTA ATC-3′ |
| MGT | 18 mer | 5′-GAT GAG TCC TGA GTA AGT-3′ |
| MAT | 18 mer | 5′-GAT GAG TCC TGA GTA AAT-3′ |
| MATA | 19 mer | 5′-GAT GAG TCC TGA GTA AAT A-3′ |
| MCAG | 19 mer | 5′-GAT GAG TCC TGA GTA ACA G-3′ |
| MATA | 19 mer | 5′-GAT GAG TCC TGA GTA AAT A-3′ |
| MTTT | 19 mer | 5′-GAT GAG TCC TGA GTA ATT T-3′ |
| MCT | 18 mer | 5′-GAT GAG TCC TGA GTA ACT-3′ |
| P-GC | 23 mer | 5′-GAC TGC GTA CAT GCA GCT GCA GC-3´ |
| PCA | 23 mer | 5′-GAC TGC GTA CAT GCA GCT GCA CA-3´ |
| PGAT | 24 mer | 5′-GAC TGC GTA CAT GCA GCT GCA GAT-3´ |
