# Supplementary material for: Identification of genes differentially expressed during interaction of Mexican lime tree infected with "Candidatus Phytoplasma aurantifolia"
Source: BMC Microbiol. 2011 Jan 1;11:1. doi: 10.1186/1471-2180-11-1 (PMC3271359; doi:10.1186/1471-2180-11-1)
Supplement: Additional File 3 — Primer sequences used for Real-Time PCR analysis. [file 1471-2180-11-1-S3.DOC]

Additional File 3. Primer sequences used for Real-Time PCR analysis

| **Primer Name** | **Annealing temperature (°C)** | **Sequence** |
| --- | --- | --- |
| 18s forward | **56** | 5' GCG GTA ATT CCA GCT CCA A 3' |
| 18s reverse | **56** | 5' TAA TTT CTT CAA AGT AAC AGC G 3' |
| F Serine protein kinase | **60** | 5' GTA CCA ATT CGT CGA AGG AC 3' |
| R Serine protein kinase | **60** | 5' TCT CTG GTA GGA ATA A GT C 3' |
| F APG5 | **61** | 5' GCG TAC CAA TTC GTC GTC 3' |
| RAPG5 | **61** | 5' GAG TCC TGA GTA AT GAA 3' |
| F RNA polymerase | **60** | 5' GAG CAG CCA TTT GAT CCC C 3' |
| R RNA polymerase | **60** | 5' CGC TGC ATA GAT TAG GC 3' |
| F Importin β 3 | **61** | 5' TAC TGA CTC AAC TGC GCG 3' |
| R Importin β 3 | **61** | 5' CGC AGT CAT GAT GAG TCC 3' |
